# Unusual neurologic manifestations of Vogt-Koyanagi-Harada disease: a systematic literature review

**DOI:** 10.1186/s12883-022-02569-6

**Published:** 2022-02-04

**Authors:** Moussa Toudou-Daouda, Abdoul Kadir Ibrahim-Mamadou

**Affiliations:** 1 Department of Neurology, National Hospital of Niamey, PO Box 238, Niamey, Niger; 2 Department of Medicine and Medical Specialties, Regional Hospital Center of Dosso, Dosso, Niger

**Keywords:** Nervous system, Vogt-Koyanagi-Harada disease, unusual neurologic manifestations

## Abstract

**Background and Purpose:**

The usual neurologic manifestations of Vogt-Koyanagi-Harada (VKH) disease include aseptic meningitis and headaches. We performed the present study to review all unusual neurologic manifestations reported in VKH disease to summarize them.

**Methods:**

A literature search was performed in the English language on Scopus and Medline via PubMed from 1946 to July 31, 2021, by using the following terms: “Vogt Koyanagi Harada disease” OR “VKH disease” AND “Brain” OR “Spinal cord” OR “CNS” OR “Central nervous system” OR “Neurologic” OR “Peripheral nervous system” OR “Polyneuropathies. Our inclusion criteria were unusual neurologic manifestations of VKH disease.

**Results:**

Our literature search yielded 417 total articles (PubMed = 334, Scopus = 83) from which 32 studies comprising 43 patients (22 men and 21 women, of which 62.8% were younger than 50 years) were included in this systematic literature review. Regarding the study design, all studies were case reports and published between 1981 and 2021. CNS involvement was the most reported (93%) in VKH disease. Peripheral nervous system involvement represents 7% of cases. The cerebral lesions were parenchymal inflammatory lesions in the white matter or posterior fossa with or no contrast enhancement (16.3%), leptomeningitis (9.3%), pachymeningitis (7%), meningoencephalitis (2.3%), ischemic stroke (4.6%), hemorrhagic stroke (2.3%), transient ischemic attack (2.3%), and hydrocephalus (2.3%). The optic nerve lesions were anterior ischemic optic neuropathy (20.9%) and optic neuritis (9.3%). Concerning spinal cord lesion, it was mainly myelitis (14%).

**Conclusion:**

This systematic literature review provides a summary of the different unusual neurologic manifestations reported in VKH disease.

## Background

Vogt-Koyanagi-Harada (VKH) disease is characterized by bilateral ocular involvement associated with extraocular manifestations such as neurological (related to aseptic meningitis: headache, neck and back stiffness), auditory (tinnitus, hearing loss, and vertigo), and integumentary (alopecia, poliosis, and vitiligo) [1]. VKH disease is a rare multisystemic autoimmune disease, mediated by T cells directed against melanocytes strongly present in the eye (choroids), inner ear, meninges, and the integumentary system [2, 3]. This disease affects mainly patients aged between 20 and 50 years, females (with a female/male ratio of 2:1), Asians, Native Americans, and Hispanics [2]. The origin of this disease remains unknown. The role of genetic factors has been recognized in the pathogenic mechanisms of VKH disease due to its strong association with certain HLA antigens [1, 4, 5]. According to the data from a systematic review and meta-analysis, HLA-DRB1*0404, HLA-DRB1*0405, and HLA-DRB1*0410 are risk sub-alleles for VKH disease [6]. VKH disease occurs in people with a genetic predisposition who are exposed to one or more environmental triggers. Infectious agents such as Epstein-Barr virus and cytomegalovirus are the mains environmental triggers reported [7, 8].

The usual neurologic manifestations of VKH disease include aseptic meningitis and headaches [9]. However, unusual neurologic manifestations had been reported in VKH disease. We performed the present systematic literature review (SLR) to summarize the different unusual neurologic manifestations reported in VKH disease.

## Methods

### Study design

The present study is a SLR focused on the unusual neurologic manifestations of VKH disease. The review protocol was not previously registered. We conducted this SLR according to the recommendations of the Preferred Reporting Items for Systematic Reviews and Meta-Analyses (PRISMA) statement. All articles included in this SLR are referenced.

### Search strategy

To carry out this SLR, a literature search was performed on Scopus and Medline via PubMed from 1946 to July 31, 2021. In both electronic databases, the literature search was performed by using the following terms: “Vogt Koyanagi Harada disease” OR “VKH disease” AND “Brain” OR “Spinal cord” OR “CNS” OR “Central nervous system” OR “Neurologic” OR “Peripheral nervous system” OR “Polyneuropathies. The search was conducted in the English language.

### Study selection

All records identified during the literature search were independently screened by the two authors (MTD and AKIM). The first stage consisted of screening based on titles and abstracts of all identified records through the literature search to identify potentially eligible articles. The second stage consisted of screening based on the full text of all potentially eligible articles to identify articles meeting the inclusion criteria of our SLR. The sole inclusion criteria for our SLR was VKH disease associated with unusual neurological involvement. We made no restrictions on the language.

### Data extraction and analysis

We manually extracted the following data from the included studies: study authors, year of publication, country, study design, age, gender, and main results. Data extraction was completed independently by the two authors (MTD and AKIM), and any discrepancies were resolved by discussion and consensus. We reported our findings using qualitative descriptive statistics. A meta-analysis was not performed because the included studies were all case reports.

### Ethics statement

Ethics approval and written informed consent were not required for this SLR because all the data were extracted from public access databases and no primary data were collected or generated during the review process.

## Results

**Fig. 1:**
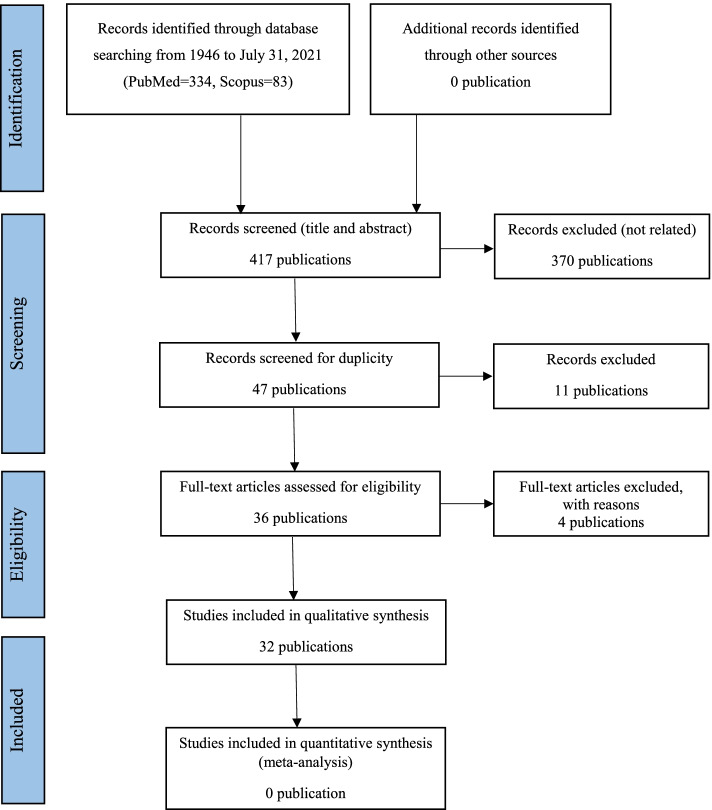
Systematic literature review flowchart

The studies selection process was showed in Fig. 1. Our literature search yielded 417 total articles (PubMed = 334, Scopus = 83). After reviewing titles and abstracts, 370 studies were excluded because they were unrelated to the aim of our SLR. Among the remaining 47 potentially eligible studies, 11 were excluded for duplicity. After reviewing the full texts of the remaining 36 articles, 4 studies were excluded because they reported usual neurologic manifestations of VKH disease. Eventually, 32 studies [10–41] fulfilled our inclusion criteria and were retained in our SLR.

### Study characteristics

Our SLR included a total of 32 publications, comprising 43 patients (22 men and 21 women, of which 62.8% were younger than 50 years). Regarding the study design, all studies were case reports and published between 1981 and 2021. There were 27 articles in English and 5 in Japanese. Table 1 summarizes the characteristics of the included studies.

**Table 1:** Characteristics of included studies

Year	First author	Study Design	Country	Sex/Age (years)	Presenting Neurological Symptoms and Signs	Neurologic Manifestations Reported	Diagnostic criteria
2021 [18]	Yu et al.	CR	South Korea	M/43	Paraplegia, sensory deficit in both lower extremities, positive Babinski’s sign, dysuria	Longitudinal myelitis	Complete VKH disease according to Revised Diagnostic Criteria [9]
2020 [11]	Patyal et al.	CR	India	F/28	–	AION	Probable VKH disease [9]
2020 [30]	El Beltagi et al.	CR	U.S.A	M/38	–	Leptomeningitis of the cerebellar folia	Probable VKH disease [9]
2019 [29]	Le et al.	CR	Australia	F/69	Ataxia of the 4 limbs	Medial temporal lobes leptomeningitis	Incomplete VKH disease [9]
2018 [26]	Pellegrini et al.	CR	Italy	F/42	–	Unilateral neuroretinitis	Complete VKH disease [9]
2017 [22]	Algahtani et al.	CR	Saudi Arabia	F/39	Dysarthria, confusion, and status epilipticus	Hyperintense periventricular lesions mimicking multiple sclerosis	Incomplete VKH disease [9]
2016 [32]	Valenzuela et al.	CR	U.S.A	M/32	–	Pachymeningitis along the clivus	Incomplete VKH disease [9]
2014 [37]	Vergaro et al.	CR	Italy	M/12	Choreic movements, unsteady gait	Ischemic stroke	Incomplete VKH disease [9]
2014 [19]	Sheriff et al.	CR	U.S.A	F/58	Peripheral facial palsy, hypoglossal nerve dysfunction, facial hypoesthesia, hemiataxia	Diffuse pachymeningitis with cerebellopontine angle inflammatory lesion	Incomplete VKH disease [9]
2013 [16]	Gu et al.	CR	China	F/50	Tetraparesis, positive bilateral Babinski’s sign	Acute myelitis	Incomplete VKH disease [9]
2013 [24]	Kales et al.	CR	Turkey	M/27	–	Hyperintense lesion in the periventricular deep white matter	–
2013 [34]	Naeini et al	CR	Iran	F/57	Disturbances of consciousness	Encephalopathy with hyperintensity in the right temporal, both frontal and right parietal lobes	Incomplete VKH disease [9]
2012 [28]	Loh Y	CR	U.S.A	M/35	Severe vertigo, bidirectional gaze-evoked nystagmus	Basilar leptomeningitis	–
2011 [27]	Lohman et al	CR	U.S.A	M/28	–	Leptomeningitis of the cerebellar folia and the interpeduncular fossa	Probable VKH disease [9]
2010 [17]	Tang et al	CR	China	F/16	Paraparesis, positive bilateral Babinski’s sign	Acute longitudinal myelitis	Incomplete VKH disease [9]
2010 [31]	Han et al	CR	South Korea	F/54	–	Anterior temporal lobes pachymeningitis	Incomplete VKH disease [9]
2009 [38]	Baheti et al	CR	India	M/26	Gaze-evoked nystagmus, bilateral upper and lower limb incoordination, gait ataxia	Cerebellar hemorrhagic stroke	Complete VKH disease [9]
2009 [10]	Nakao et al	CR	Japan	F/79	–	AION	Incomplete VKH disease [9]
				M/65	–	AION	Incomplete VKH disease [9]
				M/64	–	AION	Incomplete VKH disease [9]
				M/63	–	AION	Incomplete VKH disease [9]
				F/54	–	AION	Incomplete VKH disease [9]
				M/70	–	AION	Incomplete VKH disease [9]
2009 [15]	Dahbour SS	CR	Jordan	F/37	Paraparesis, positive Lhermitte’s sign, positive Romberg’s test, urinary urgency	Acute myelitis	Complete VKH disease [9]
2009 [20]	Hashimoto et al	CR	Japan	M/28	One-and-a-half syndrome, facial nerve palsy, stuporous, palate paresis	Brainstem encephalitis	Complete VKH disease [9]
2007 [25]	Rajendram et al	CR	U.S.A	F/35	–	Optic neuritis	Probable VKH disease [9]
				F/35	–	Optic neuritis	Probable VKH disease [9]
				M/25	–	Optic neuritis	Probable VKH disease **[**9**]**
2007 [40]	Yamamoto et al	CR	Japan	F/43	Urinary incontinence, disturbance of consciousness	Hydrocephalus	–
2006 [13]	Abematsu et al	CR	Japan	F/51	–	AION	–
2001 [33]	Najman-Vainer et al	CR	U.S.A	M/43	Weakness and decreased sensitivity of the lower limbs, abolition of Achilles tendon reflexes and diminution of the patellar tendon reflexes	Guillain-Barré syndrome	Incomplete VKH disease [9]
				F/63	Weakness of the 4 extremities and facial muscles, areflexia	Guillain-Barré syndrome	Incomplete VKH disease [9]
				M/48	Weakness of the 4 extremities, bilateral facial nerve palsy, areflexia	Guillain-Barré syndrome	Probable VKH disease [9]
2000 [35]	Kamondi et al	CR	Hungary	F/36	Somnolence, hemiparesis, supranuclear hypoglossal paresis	Meningoencephalitis	–
1999 [12]	Yokoyama et al	CR	Japan	M/68	–	AION	–
1995 [23]	Osaki et al	CR	Japan	M/57	Truncal ataxia	Contrast enhancement of both the uveas and the cerebellar vermis	–
1995 [39]	Ryan et al	CR	U.S.A	F/59	Several episodes of weakness and numbness of left or right-sided	Transient ischemic attacks, bilateral carotid stenosis	–
1992 [21]	Ikeda et al	CR	Japan	F/40	Unconsciousness, meningeal stiffness, facial muscles weakness	Inflammatory lesions of pons, cerebellum, temporoparietooccipital regions, caudate nucleus, and putamen	–
1989 [41]	Hiraki et al	CR	Japan	M/32	Gait disturbance, limb and truncal ataxia	–	–
				M/22	VIIth, VIIIth, IXth, and Xth cranial nerve palsies	–	–
1989 [36]	Nitta et al	CR	Japan	M/45	Vertigo, vomiting, positional nystagmus, diplopia, Horner’s syndrome on the right side, right facial palsy, palsy of the soft palate on the right side	Cerebellar infarction	–
1981 [14]	Lubin et al	CR	U.S.A	F/22	Paraparesis, alteration at all modes of the sensitivity of the lower limbs	Myelitis	–
				M/21	Ataxic gait, positive Romberg’s test, urinary disorders (urinary retention)	Myelitis	–

*CR* indicates case report, *F* female, *M* male, *AION* anterior ischemic optic neuropathy, *VKH disease* Vogt-Koyanagi-Harada disease

### Unusual neurologic manifestations of VKH disease

Table 1 summarizes the main unusual neurological manifestations in this SLR. CNS involvement was the most reported (93%) in VKH disease. Only reported by one study [33], peripheral nervous system involvement represents 7% of cases. Among the CNS involvement (40 cases), cerebral lesions represented 52.5% of cases (21/40), followed by the optic nerve lesions (13/40 = 32.5%) and the spinal cord (6/40 = 15%).

The cerebral lesions were parenchymal inflammatory lesions in the white matter or posterior fossa with or no contrast enhancement (16.3%) [19–24, 34], leptomeningitis (9.3%) [27–30], pachymeningitis (7%) [19, 31, 32], meningoencephalitis (2.3%) [35], ischemic stroke (4.6%) [36, 37], hemorrhagic stroke (2.3%) [38], transient ischemic attack (2.3%) [39], and hydrocephalus (2.3%) [40].

The optic nerve lesions were anterior ischemic optic neuropathy (20.9%) [10–13] and optic neuritis (9.3%) [25, 26]. Concerning spinal cord lesion, it was mainly myelitis (14%) [14–18].

## Discussion

In the present SLR, we found that unusual neurologic manifestations of VKH disease are rare, and all reported studies are case reports. The evidence level of nervous system involvement or neurologic manifestations of VKH disease is moderate to high quality. In the majority of studies included in this SLR, the patients had benefited from an exhaustive exploration that had permitted ruling out other conditions such as Behçet’s disease, neuromyelitis optica spectrum disorder, tuberculosis or sarcoidosis. All included patients had an established diagnosis of VKH disease. The patients with ischemic stroke [36, 37] had undergone a work-up that had permitted ruling out a cardiac or atherosclerotic origin.

VKH disease is a systemic autoimmune disorder affecting melanocyte-rich tissues, such as the eyes, inner ear, meninges, and skin [2, 3]. The unusual neurologic manifestations of VKH disease are various and dominated by cerebral involvement, like inflammatory parenchymal lesions. The precise pathophysiological mechanism by which VKH disease leads to cerebral or spinal cord involvement is unclear. The brain, optic nerves (prolongation of the brain), and the spinal cord are surrounded by meninges. These meninges contain strongly melanocytes which are T cell targets in VKH disease [42]. That could explain the cerebral involvement, optic nerves (optic neuritis), and the spinal cord observed in VKH disease. Concerning anterior ischemic optic neuropathy (AION), the pathophysiological mechanism of its occurrence is uncertain. The vascularization of the optic disc is organized as follows: 1) the lamina cribrosa region is supplied by centripetal branches directly from the short posterior ciliary arteries (PCAs) or from the circle of Haller and Zinn formed by the short PCAs (when that is present), and 2) the prelaminar region is supplied by the fine centripetal branches from the peripapillary choroidal vessels [43]. Severe uveitis with choroidal involvement causes inflammatory infiltration of the peripapillary choroidal vessels with a high risk of their obliteration that could explain the occurrence of the AION in VKH disease.

Magnetic resonance imaging (MRI) is the preferred imaging technique for detecting brain or spinal cord lesions in patients with VKH disease and helps in the differential diagnosis of VKH disease with multiples sclerosis. MRI can detect the meningeal inflammatory process in patients with VKH disease by showing pachymeningeal or leptomeningeal enhancement.

Peripheral nervous system involvement found in this SLR was Guillain-Barré syndrome [33]. The pathophysiological mechanism of Guillain-Barré syndrome in VKH disease is not well known. Since melanocytes and Schwann cells (myelin-producing cells) had the neural crest as a common embryologic origin [44], it is easy to suppose that a disease involving melanocytes (such as VKH disease) can cause peripheral nervous system involvement.

### Limitations

The main limitation of this SLR is that it is mainly based on case reports. However, VKH disease is a rare condition and its unusual neurologic manifestations are even rarer, which would explain the small number of reported cases in the literature.

## Conclusions

This SLR summarizes the findings of existing studies on unusual neurologic manifestations of VKH disease and provided data on the pathophysiological mechanisms of the occurrence of these neurologic manifestations during this disease. Nervous system involvement or neurologic manifestations of VKH disease have been well documented in patients included in this SLR. To our knowledge, our study is the sole systematic review performed on the unusual neurologic manifestations of VKH disease.

## Data Availability

NA

## Abbreviations

AION: Indicates anterior ischemic optic neuropathy
CNS: Central nervous system
MRI: Magnetic resonance imaging
SLR: Systematic literature review
VKH: Vogt-Koyanagi-Harada
